# Neck muscle twitch properties are associated with constraint on drum speed in woodpeckers, but not drum length

**DOI:** 10.1242/jeb.251289

**Published:** 2025-12-03

**Authors:** Nicholas D. Antonson, John G. Capano, Matthew J. Fuxjager

**Affiliations:** Department of Ecology, Evolution and Organismal Biology, Brown University, Providence, RI 02912, USA

**Keywords:** Motor constraint, Multicomponent signaling, Display performance, Signal modularity, *Dryobates pubescens*

## Abstract

Animal displays are often limited by the properties of the muscles that generate them. Here, using *in situ* muscle stimulation, we investigated the twitch properties of the longus colli ventralis (LCv), a primary muscle used to protract the head and neck during territorial drumming displays in woodpeckers. Specifically, we tested LCv twitch kinetics and endurance in a manner that simulates drum speed (beats s^−1^) and length (total beats), two signal features that can evolve independently of each other. We identified a maximum muscle contraction rate that may represent a physiological constraint relevant to drumming speed, but no relevant constraint on the repetition of contractions that might affect drum length. This suggests that twitch properties may differentially affect display components. Broadly, our findings highlight how certain display features may freely diversify independent of others owing to physiological limits, while pointing to the way complex signals can evolve under partial performance constraints.

## Teaser

**Summary:** Distinct muscular constraints affect performance in woodpecker drumming. Twitch kinetics limit the contraction–relaxation frequency necessary for fast drum speeds, but there was no analogous constraint on muscle twitch properties for longer drum lengths.

## INTRODUCTION

For most animals, skeletal muscle performance plays a critical role in the production of elaborate communication signals (Fuxjager et al., 2016; Katz and Hale, 2017; Schaeffer et al., 1996). One reason is that muscle limits how these signals are generated, thereby acting as a powerful force of constraint (Byers et al., 2010; Garland et al., 2022; Logue and Bonnell, 2023; Podos, 2022). For signals with multiple functionally salient features, these effects can be quite complex – indeed, some signal features may be ‘co-limited’ by singular attributes of muscle performance, whereas other sets of features might be each limited by distinct attributes of muscle performance (Mitoyen et al., 2019; Partan and Marler, 2005). An example of the former comes from work in manakin birds, which suggests that contraction–relaxation cycling dynamics impose constraints on speed and length of wing-snap displays used for courtship (Fuxjager et al., 2022; Miles et al., 2018a). Conversely, work in songbirds suggests that constraints on signal performance are ‘partitioned’ across different levels of the motor system, ranging from the muscles within the vocal organ to neural systems that control these tissues (Alward et al., 2018; Schmidt and Goller, 2016; Wild, 2004). Such partitioning may reduce performance trade-offs by giving rise to structural modularity, wherein each signal component can evolve independently (Hebets et al., 2016). Although a growing body of literature examines how muscle physiology limits one display parameter, far fewer studies have addressed whether multiple features are constrained at the same or different physiological sites (Katz and Hale, 2017; Syme and Josephson, 2002).

Woodpecker drum displays provide a tractable system to address this topic. Drumming involves repeated hammering of the bird's bill against a substrate, generating a loud staccato-like acoustic signal used for territorial interactions (Schuppe et al., 2021). Drums vary temporally in speed (beats s^–1^) and length (total beats), with behavioral studies showing that faster and longer drums generally signal greater territorial prowess (Schuppe et al., 2016; Schuppe and Fuxjager, 2018). Further, individuals tightly adhere to drumming at their own species' typical speed and length, with both components being positively associated with competitive prowess (Dodenhoff et al., 2001; Miles et al., 2018b; Moody et al., 2022, 2025).

Physiologically, the longus colli ventralis (LCv) – a hypertrophied neck muscle – drives head protraction in a one-to-one manner for each strike within a drum (Antonson et al., 2025; Jenni, 1981). This tissue also exhibits molecular signatures to support rapid twitch kinetics, implying a specialized role in powering fast drum frequencies (Schuppe et al., 2018, 2022b). Importantly, behavioral observations suggest that speed is mechanistically constrained: most woodpeckers cannot exceed their species-typical drum rate, and even simulated territorial intrusions that push speed higher fail to elicit matching speed outputs above those characteristic to the species (Moore et al., 2022; Schuppe and Fuxjager, 2018). However, it remains unclear whether the same muscle properties that may constrain drum speed also limit drum length, which seems plausible because repetitive displays often face length constraints (Dos Santos et al., 2023; Miles et al., 2018a; Remage-Healey and Bass, 2005). In woodpeckers, drum length varies widely, even within a species (Miles et al., 2018b; Moody et al., 2022), yet we know little about how physiological factors may cap this trait.

Here, we tested whether LCv twitch properties constrain this muscle's performance as it relates to drumming in male downy woodpeckers (*Dryobates pubescens*). We did so to assess whether these twitch properties have the potential to generate constraints that explain species-level differences in drumming. Specifically, we considered the muscle's relaxation ability in response to repeated twitch stimuli that vary in frequency and number as metrics relative to drum speed and length, respectively. We hypothesized that rapid LCv twitch kinetics set upper physiological limits on maximum attainable drum speeds (beats s^–1^), given the necessity of this muscle to relax fully to initiate subsequent contractions effectively (Josephson, 1993). Additionally, we tested how force production changes across repeated muscle contractions, hypothesizing that relative force diminishes later in stimulation trains (i.e. fatigue onset and accumulation) to restrict the total beat production (Enoka, 2012). Finally, constraints might be interactive, restricting muscle relaxation and force in response to a combination of increased twitch frequency and twitch number or vice versa. Alternatively, relaxation or force production may be affected by either increasing the stimulus length or speed, but not both.

To evaluate all these possibilities, we used an *in situ* stimulation approach (Fuxjager et al., 2016, 2017; Mencio et al., 2017; Sica and McComas, 1971; Tobias and Kelley, 1987) that attempts to mirror how the LCv would be activated to power drumming, while experimentally varying certain parameters of this activation (stimulation rates, total stimulation pulses).

## MATERIALS AND METHODS

### Animals and ethical note

Reproductively active adult male downy woodpeckers [*Dryobates pubescens* (Linnaeus 1766)] were captured (*n*=5) via mist netting with conspecific playback from May to August 2023 and 2024 near Providence, RI, USA. Birds were transported to Brown University for *in situ* muscle stimulation assays (see below). Upon completion of stimulations, we banded and released individuals back to their capture sites. All methods were approved by the Institutional Animal Care and Use Committee of Brown University (no. 19-09-006), the United States Fish and Wildlife Service, and the Rhode Island Department of Environmental Management. We verified that all birds were in breeding condition (e.g. no molt, enlarged gonads; Hahn and Ball, 1995).

### Stimulation design

The focus of our study was the longus colli ventralis (LCv) neck muscle, which protracts the head during drumming (Antonson et al., 2025). We designed stimuli that we would expect to mirror a drum; namely, a train of electrical stimuli that occur at roughly the same frequencies at which birds produce drums. First, we generated these ‘drum instructions’ to represent (i) average speed (mean±s.d.=15.995±0.949 beats s^−1^) and length (mean±s.d.=16.5±4.087 beats) for downy woodpeckers, (ii) 1.5 and 3 s.d. faster-than-average speeds, (iii) 1.5 and 3 s.d. longer-than-average lengths, (iv) combined increases in speed and length, and (v) drums characteristic of two other similarly sized, distantly related, higher-performance woodpecker species: (1) Japanese pygmy woodpeckers (*Yungipicus kizuki*; 8 beats long at 38 beats s^−1^) and (2) blood-colored woodpeckers (*Veniliornis sanguineus*; 29 beats long at 26 beats s^−1^) (Fig. S1). These species were chosen because they represent extremes in drum speed and length, without confounding the experimental design with the effects of body size.

Stimulation trains were designed such that one stimulation pulse was coordinated in a one-to-one manner with drum strikes (*sensu* Antonson et al., 2025). All downy woodpecker stimulations were designed such that they had the same average decelerating linear model slope (*m*=0.485). These stimuli enabled us to test whether LCv performance imposes ‘co-localized’ constraint on both speed and length or only one component. Additional details about stimulation profiles are provided in the Supplementary Materials and Methods.

### *In situ* muscle stimulation

We recorded LCv twitch properties using a nonterminal *in situ* approach detailed elsewhere (Fuxjager et al., 2016, 2017; Miles et al., 2018a). The method closely resembles preparations described in other studies that explore twitch dynamics of anuran laryngeal muscles (Leininger et al., 2015; Potter et al., 2005; Tobias and Kelley, 1987; Zornik and Yamaguchi, 2011), avian syringeal muscles (Mencio et al., 2017), rat gastrocnemius muscle (de Haan, 1998) and human extensor hallucis brevis muscle (Sica and McComas, 1971). Briefly, birds were anesthetized with isoflurane (1–4% in O_2_), placed on a heating pad, and positioned to make a minor incision (>0.5 cm) and expose the LCv. We inserted small electrode pins into the muscle belly and secured a stainless-steel hook to the distal portion of the LCv. Additionally, the LCv was connected via a hook to a force transducer (FT03, Grass Technologies) through a monofilament line and drawn taut to the muscle's *in vivo* resting length, allowing muscle shortening during each twitch but preventing large excursions or uncontrolled strain (*sensu* Sica and McComas, 1971). Stimulation trains to induce twitches were produced at 0.5 mA submaximal stimulation by an isolated high-power stimulator (A-M Systems Model 4100). Throughout stimulation trials, normal saline was applied to prevent desiccation. After each experiment, we closed the incision with suture and tissue adhesive and allowed the bird to fully recover before release.

We chose sub-maximal, near-isometric *in situ* stimulation because it more closely preserves the LCv's origin-insertion geometry, passive connective-tissue tension, body temperature and vascular supply, while still allowing precise electrical control of activation (Fuxjager et al., 2016; Miles et al., 2018a). Because the muscle was free to shorten slightly under load, our setup likely engaged shortening dependent deactivations, a phenomenon in which active shortening reduces Ca^2+^ sensitivity and accelerates relaxation (Josephson, 1975, 1999; Marsh, 1990). This would effectively decrease the twitch durations leading to an increase in percent relaxation in response to a given twitch in a series (one of our main performance metrics in this study).

During repetitive activation, muscles can accumulate metabolic byproducts (e.g. inorganic phosphate, H^+^) and experience excitation–contraction coupling failure, which together reduce the ability to generate and sustain force (Enoka, 2012; Miller et al., 1993). Such fatigue often emerges more quickly during high-frequency or long-duration trains of stimulation, leading to a progressive decline in twitch amplitude. Thus, if longer trains of drumming stimuli induce a decrease in normalized force output, this would suggest that fatigue constrains the total attainable drum length.

During real drumming, the muscle shortens against inertial and elastic loads, conditions that typically slow relaxation kinetics relative to our semi-isometric testing (Johnson et al., 1993). Although fully *in vitro* fiber or bundle assays can deliver maximal activation and greater experimental control, they can greatly reduce tendon elasticity, alter resting length, and disrupt the natural series-compliance, which would also typically slow relaxation kinetics (Josephson, 1993). Taken together, our *in situ* protocol trades off some experimental control to strike a balance with biologically relevant contractile dynamics, and provides a conservative upper-bound estimate of the contraction–relaxation cycling of the LCv (Holt et al., 2014; Tijs et al., 2021).

### Data collection, percent relaxation and force decline

Muscle tension (contraction force) was amplified (5000–10,000) and low-pass filtered (3000 Hz) with an AC/DC strain gage amplifier (Model P122, Grass Technologies), then digitized at 100 kHz (LabChart, AD Instruments). We focused our analysis on the percentage of full relaxation achieved by the LCv between successive stimulation pulses within each train and the relative force produced by each of these twitches.

**Fig. 1.**
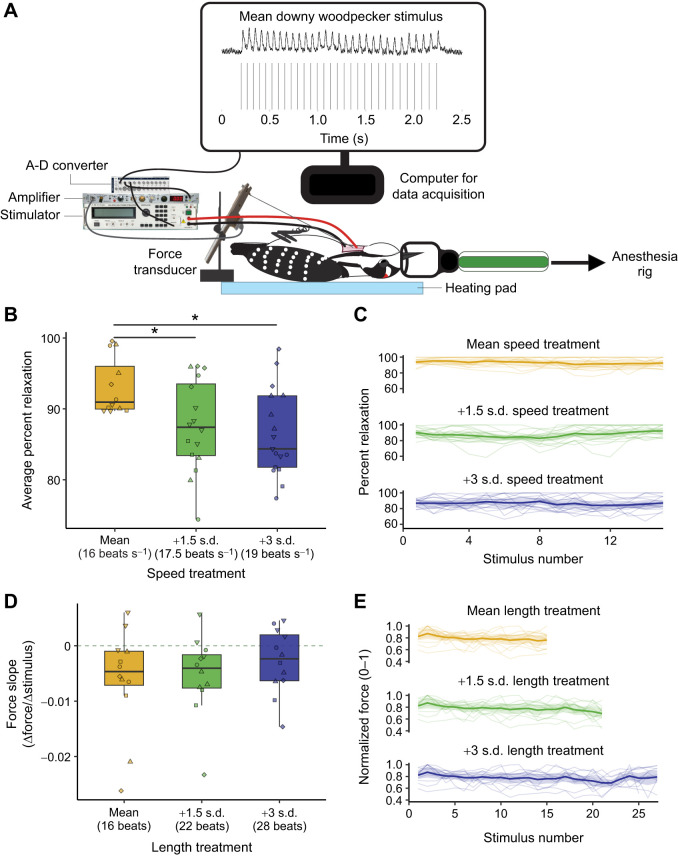
***In situ* muscle stimulation on the longus colli ventralis (LCv) with stimulations trains matching those of downy woodpecker drums.** (A) Experimental setup. Computer screen shows raw force transducer data from the LCv (top) in response to stimulation trains (bottom) at the average downy woodpecker drum speed. (B) Average LCv percent relaxation when stimulated at mean (yellow), +1.5 s.d. (green) and +3 s.d. (blue) frequencies above mean speed. (C) Percent relaxation changes across each stimulation train for each speed treatment at mean drum length. (D) Slopes representing the change in normalized force when stimulated for the mean (yellow), +1.5 s.d. (green) and +3 s.d. (blue) of the mean drum length. Grey line denotes slope=0. (E) Normalized force changes across each stimulation train for each length treatment. Boxplots show data quartiles (thick line=median), with jittered raw data (2–5 replicates per bird, individuals depicted by common shapes). *Significant *post hoc* tests (*P*<0.05). Spaghetti plots show mean and 95% confidence intervals on top of individual trains connected continuously.

Percent relaxation was calculated by comparing each twitch peak to its subsequent trough relative to baseline (see Fig. 1A and Fig. S2). Thus, complete muscle relaxation (100% relaxation) occurred when contraction-induced tension on the force transducer was completely relieved (i.e. return to baseline). By contrast, partial muscle relaxation (<100%) occurred when contraction-induced tension on the force transducer was only partially relieved (i.e. no return to baseline). Contractions were never fully tetanic between stimulation pulses, so it was always possible to resolve percent relaxation.

For each train, peak twitch force was extracted as the difference between the baseline and force peak for each twitch. Baseline force was estimated from the same time windows immediately before and after the train. We used within-train changes in normalized force as an indicator of muscle fatigue as calculated using the β coefficient (linear slope). The resulting slope provided a robust, unitless index of whether force declined (negative slope) or was maintained (slope≈0) across the train, which is more robust to variability in single twitches than point estimates and directly reflects fatigue-related trends. A systematic decline in force across successive twitches would be consistent with fatigue observed in other small muscles (Miller et al., 1993). Conversely, stable force across longer trains would indicate that the LCv can resist fatigue over the range of contractions typical for natural drumming. This approach provides a functional test of whether fatigue at the muscle level could act as a constraint on drum length.

### Validation of LCv performance

To verify that repeated stimulations did not lead to a direction reduction in twitch performance of the LCv beyond the effects of the stimuli, we compared relaxation values from identical stimulation trains given at the start and end of the experiment for each bird (Fuxjager et al., 2016; Miles et al., 2018a). There was no significant difference between these trials (*t*_20.479_=0.353, *P*=0.728), indicating no progressive decline in muscle capacity across the entire experimental stimulation program.

### Statistical analysis

We used linear mixed-effects models (R package lme4, α=0.05) to analyze average percent relaxation and change in force as a function of drum speed or length treatment, with individual ID as a random intercept. This random intercept accounts for multiple trials of each stimulus for the *n*=5 birds and allows us to statistically account for variation across stimulation train trials. Graphs display the individuals by the shape of each datapoint and the number of trials within each individual by the number of each shape. Estimated marginal means with 95% confidence intervals and Tukey *post hoc* tests compared baseline (mean) versus elevated (1.5, 3 s.d. above mean) speed or length. To assess speed–length synergy, we tested for reduced relaxation and declining force production at higher speeds nested within extended lengths and vice versa. Finally, we performed *post hoc* tests for differences between the stimulation trains approximating the drums of our similar-sized species (Miles et al., 2018b). Further details on the statistical analysis can be found in the Supplementary Materials and Methods.

## RESULTS AND DISCUSSION

Our results show that twitch properties of the downy woodpecker LCv are limited in ways that might constrain some – but not all – features of drum behavior (Fig. 1B,C). When the LCv was stimulated to produce twitches that correspond to rates exceeding the species' natural drum frequency, percent relaxation dropped significantly (*F*_2,38_=8.82, *P*<0.001), implicating peripheral limits on rapid contraction–relaxation cycling for high-frequency head protraction. Indeed, *post hoc* tests showed significantly reduced percent relaxation when the LCv was stimulated at frequencies 1.5 s.d. (*P*=0.003) and 3 s.d. (*P*=0.001) faster than mean drum speed, whereas these latter two treatments did not differ from each other (*P*=0.769) (Table S1). Because each drum strike is likely preceded by a discrete LCv contraction (Antonson et al., 2025), the muscle's ability to fully relax between contractions is presumably tied to drum frequency. Put another way, incomplete relaxation between contractions may limit the ability of the LCv to produce sufficient force during subsequent contractions to actuate the head for drumming (Enoka, 2012; Josephson, 1993; Marsh and Bennett, 1985).

Next, we measured how force produced by LCv contractions changed in response to stimulating the muscle at or above the downy woodpecker's natural length range, while maintaining mean speed (Fig. 1D,E). Contrary to our hypothesis that the muscle may fatigue across longer stimulation trains, we found no difference in the slope of force production amongst treatments (*F*_2,29_=0.15, *P*=0.860; Table S2). Further, confidence intervals for the average slope of each length treatment overlapped zero (Table S3), which is consistent with the view that force production does not decline in response to longer stimulation trains. Thus, twitch properties of the LCv may not constrain drum length, even though comparative- and species-level evidence shows that woodpeckers do not perform indefinitely long drumrolls (Miles et al., 2018b; Moody et al., 2022). Many vertebrate courtship and/or agonistic signals are subject to endurance limits (Dos Santos et al., 2023; Miles et al., 2018a); thus, drum length in woodpeckers may be limited by alternate sites within the motor apparatus. The brain regions dLN, dAN and dA, which appear to be putative analogues of oscine song control nuclei (Schuppe et al., 2022a), could regulate the overall duration of the drum, whereas local circuits in the hindbrain might set the fundamental rhythmic pattern (Kao and Brainard, 2006; Long and Fee, 2008). Hormonal mechanisms may also selectively extend drum duration through length without shifting speed (Knapp et al., 1999; Remage-Healey and Bass, 2005). Indeed, rapid hormone fluctuations can modulate signal repetition rate or duration in many vocal and gestural displays (Anderson et al., 2021; Vitousek et al., 2014). Therefore, length may remain flexible and be significantly more variable than speed (Miles et al., 2018b), if it is not limited by the LCv.

**Fig. 2.**
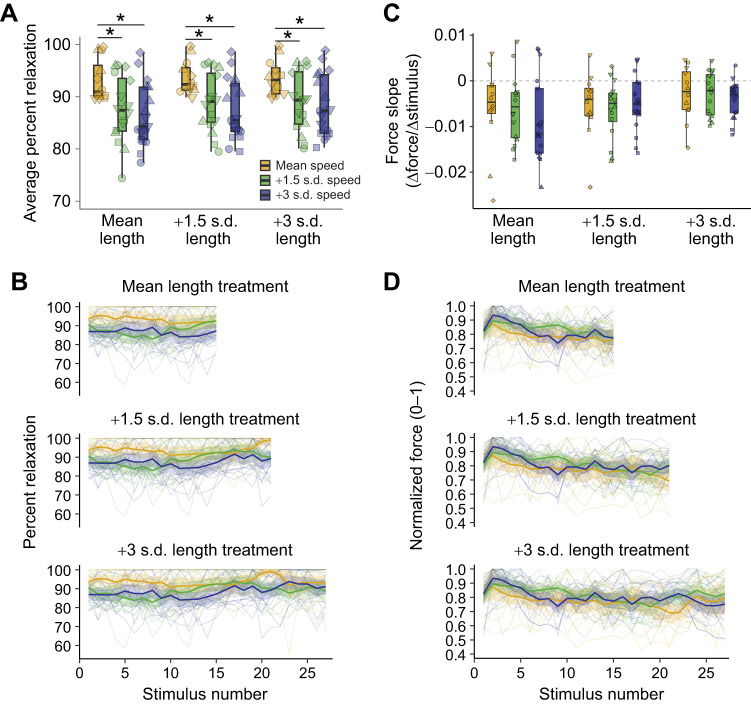
**Potential interactive effects of LCv muscle twitch properties in response to speed and length treatments.** (A) Average percent relaxation comparing speed treatments at each length treatment. (B) Spaghetti plots of percent relaxation across lengths and speeds. (C) Slope of force change across stimulation trains for each speed and length treatment combination. Grey line: slope=zero. (D) Normalized force at each stimulus in trains of different speed and length treatments. Boxplots show data quartiles (thick line=median), with jittered raw data (2-5 replicates/bird, individuals depicted by common shapes). *Significant *post hoc* tests (*P*<0.05). Spaghetti plots show mean and 95% confidence intervals on top of individual trains connected continuously.

We also tested whether the number and frequency of twitches might jointly constrain each other in a manner consistent with a speed–length trade-off in behavioral performance, as seen in some extreme displays (Alward et al., 2018; Fuxjager et al., 2013; Miles et al., 2018a). However, increasing the number of twitches (length) had no effect on average percent relaxation, and when combined with faster speed treatments, the interactive treatment did not appear to amplify the reduction in relaxation shown at the mean length treatment (*F*_4,122_=0.15, *P*=0.961; Fig. 2A; Table S1). Slopes of percent relaxation were also shallow, and their confidence intervals mostly overlapped zero (Table S4), with some combinations trending toward slight positive trajectories in the opposite direction of fatigue and no overall interaction (*F*_4,122_=2.18, *P*=0.076; Fig. 2B). By contrast, slopes of normalized force across twitches remained close to zero and did not differ significantly among speeds and across lengths (*F*_4,122_=0.61, *P*=0.659; Fig. 2C,D; Tables S2, S3), indicating that force output was stable within trains. Taken together, these analyses suggest that speed and length manipulations did not synergistically constrain relaxation or force.

**Fig. 3.**
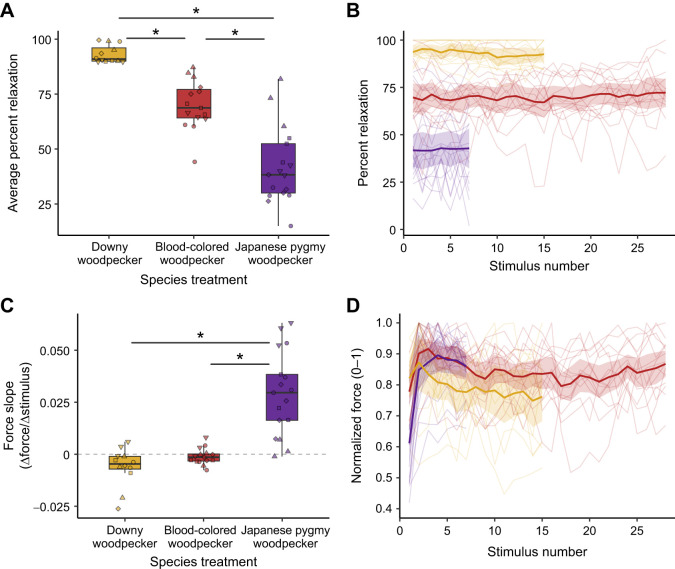
**Muscle twitch properties of the LCv in response to stimulation treatments approximating the drums of conspecific Japanese pygmy and blood-colored woodpeckers.** Species treatments are compared by (A) average percent relaxation, (B) percent relaxation across stimulation trains, (C) slope of force across stimulation trains and (D) normalized force at each stimulus in a train. Boxplots show data quartiles (thick line=median), with jittered raw data (2–5 replicates per bird, individuals depicted by common shapes). *Significant *post hoc* tests (*P*<0.05). Spaghetti plots show mean and 95% confidence intervals on top of individual trains connected continuously.

Finally, we compared relaxation and normalized force by the LCv in downy woodpeckers in response to two similarly sized high-performance species to consider muscle constraint in the context of species identity: Japanese pygmy woodpecker (i.e. +22 s.d. speed, −2 s.d. length of mean downy drum) and blood-colored woodpecker (i.e. +10 s.d. speed, +3 s.d. length) (Fig. 3). Percent relaxation of the downy LCv differed significantly among these treatments (*F*_2,37_=97.001, *P*<0.001). When stimulated to perform the Japanese pygmy or blood–colored woodpecker drum treatments, the downy LCv relaxation displayed near–complete fusion, indicating that this muscle is physiologically unable to cycle at rates as fast as these species' signals (all *P*<0.001; Fig. 3A; Table S5), though confidence intervals for all slopes overlapped zero (Fig. 3B; Table S6). These differences in twitch kinetics likely underlie some of the broad interspecific variation in drumming performances across woodpeckers (Dodenhoff et al., 2001; Miles et al., 2018b; Schuppe and Fuxjager, 2018). Further, force production did not decrease in response to the long and fast blood-colored woodpecker stimulus, and the slope of force across the stimulation train even increased in response to the short and fast Japanese pygmy woodpecker stimulus compared with the other treatments (*P*<0.001 compared with both other species). This result confirms that LCv force production in the downy woodpecker was not subject to these challenging simulated demands on speed and length (Fig. 3C,D; Tables S7, S8).

These results highlight how differential constraints, such as the twitch limits we observed here, presumably help create different evolutionary pathways for speed versus length, consistent with macroevolutionary evidence that drum length elaboration can evolve more quickly than speed within lineages (Miles et al., 2018b; Moody et al., 2022). Specifically, if selection were to favor changes in speed, the physiological ceilings we demonstrate are imposed on the LCv twitch kinetics may slow or even canalize evolutionary responses in drum speed, thereby enforcing stability around species mean speeds. By contrast, drum length in our measurement does not face a similar constraint at the LCv, and thus selection can more freely drive trait elaboration or diversification in this signal component. In this way, the distinct physiological bases of speed and length shape not only their current phenotypic expression, but also the course and pace of their evolutionary trajectories. More broadly, this pattern illustrates how partial physiological constraints foster structural modularity, in which different display components follow independent trajectories and contribute separately to signal diversification (Hebets et al., 2016; Zambre and Thaker, 2017).

### Conclusions

Physiological constraints have long been recognized as important forces shaping the evolution of communication signals (Byers et al., 2010; Zahavi, 1975). Here, we show that twitch properties of the downy woodpecker LCv may impose a cap on drum speed, but not drum length. To this end, downy woodpeckers were unable to perform muscle twitch profiles that reflect two other species with high-performance drums, suggesting that limits to contraction–relaxation cycling dynamics might help enforce species-level differences in signal performance. Together, these results suggest that the twitch dynamics underlying drumming features are limited by separate mechanisms, which implies structural modularity in signal production. Future work should focus on how the distinct genetic bases of structurally modular multicomponent signals such as drumming lead to independent selection for phenotypic traits such as speed and length.

## Supplementary Material

10.1242/jexbio.251289_sup1 Supplementary information
