# Supplementary material for: Neck muscle twitch properties are associated with constraint on drum speed in woodpeckers, but not drum length
Source: J Exp Biol. 2025 Dec 3;228(23):jeb251289. doi: 10.1242/jeb.251289 (PMC12745930; doi:10.1242/jeb.251289)
Supplement: Supplementary information [file jexbio-228-251289-s1.pdf]

Supplementary Materials and Methods

Further Details on Stimulation Design

As described above, the aim of our study was to subject the LCv of male downy woodpeckers to electrical stimulation pulses that mirror “activational” programs the brain likely sends to the muscle to actuate head and neck movements associated with drumming (Fig. S1). We determined the average downy woodpecker drum pattern (stimulus i) using freely available data on XenoCanto, an online repository for acoustic recordings of birds (https://xeno-canto.org/). Mean drum speed for this population was 15.995 beats sec-1 with 1 SD equaling 0.949, whereas mean length was 16.5 beats with 1 SD equaling 4.087 (Miles et al., 2018). Note that we used 16 beats as the average drum length and 4 beats as the SD because there are no partial beats in a drum. Downy woodpeckers produce drums that have a rhythm characterized by a linear deceleration (Miles et al., 2020); therefore, we ensured that this rhythm was preserved in the rate of electrical stimulation that made up our activation program. We did this by plotting time durations (in msec) between successive beats in a drum (i.e., the inter-beat intervals) as a function of inter-beat interval number. We then fit a linear regression to these data, calculating an average slope (m) that described the change in IBIs over time (average m=0.485, SD=±0.227). Altogether, this meant that, for all downy woodpecker stimuli described below, we ensured that speed change occurred at the rate described by m (see also Miles et al., 2020; Schuppe and Fuxjager, 2018 for further descriptions of woodpecker drum rhythm). In regards to the drums of the two woodpeckers are comparable in size to downy woodpeckers, we chose these species not just because of their drum performance profiles but also because previous studies suggest that body size constrains the evolution of drum speed, such that absolute body size may biomechanically change how a drum is performed (e.g., different lever arm lengths) (Miles et al., 2018). Both species perform drums with virtually no change in acceleration between beats of their drum, which differ from downy woodpeckers, which slow down across the drum roll. Maintaining a fast drum is likely to be more physically demanding

Journal of Experimental Biology • Supplementary information

compared to slowing down, and thus high speeds are likely to further enforce any species differences in drum performance. A recent in vivo electromyographical study suggested no evidence for changes in LCv activation timing due to impact-rebound effects that would lead to differences between the first and subsequent strikes produced in a series (Antonson et al., 2025), so we did not modify the production of the first twitch relative to the other twitches of the series.

Muscle Stimulation Justification and Further Details

We choose this non-terminal *in situ* approach because of our fundamental interest in the way muscle performance might constrain behavioural actuation; thus, in situ methods have the benefit of reflecting muscle performance under natural conditions and loads. More traditional in vitro assays of muscle mechanics can sometimes mis-estimate performance features as they relate to behaviour, potentially creating ambiguity in terms of sources of motor constraint (e.g., Elemans et al., 2008 vs Mencio et al., 2017) and animals under *in vivo* conditions often do not spontaneously perform their full behavioral repertoire in a laboratory setting.

Once the *in situ* setup was complete, we measured percent relaxation of the muscle as the maximum contraction in response to stimulus and subsequent ability to relax. All stimulation trains were performed in 3-5 replicates. Rarely a trace was too noisy to analyze in which case only two replicates were used for analysis. Sub-maximal stimulation was at sufficiently low amplitudes (0.5 mA) in the muscle belly that individual pulses caused virtually no head movement while still pulling on the force transducer line.

Further Detail on Calculating Percent Relaxation

To determine percent relaxation for the muscle, we measured the percentage at which the LCv relaxed after each stimulation pulse within each stimulation train. The baseline signal of the force transducer

Journal of Experimental Biology • Supplementary information

was calculated by averaging six peaks within a 10 msec window of the baseline noise just prior to muscle stimulation. To measure percent relaxation, we calculated (a) baseline signal just prior to each stimulation train, (b) the peak of interest – showing the extent of relaxation between stimulation pulses, and (c) the maximal force caused by each stimulation in the train. Using these points, we determined the value of full relaxation in response to each stimulation of the train, and the percentage of full relaxation actually achieved in response to the stimulation frequency (Fig. S1; Fig. 1a). We constrained percent relaxation thresholds at or below 100% (maximum relaxation) and at or above 0% (minimum relaxation, or full muscle fusion). Thus, complete muscle relaxation (100% relaxation) occurred when contraction-induced tension on the force transducer was completely relieved (i.e., return to baseline). By contrast, partial muscle relaxation (<100%) occurred when contraction-induced tension on the force transducer was only partially relieved (i.e., no return to baseline). Partial relaxation ranges as a function of stimulation frequency, and it is calculated by dividing the magnitude of the measured relaxation by the magnitude of recovery that would have been necessary for full relaxation (Fig. S2).

Statistical Analysis

Determining the effect of speed and length

Statistics were determined in R (version 4.0.2), using an α value of 0.05 for all models. To determine the influence of fixed and random effects on average percent relaxation and the slopes of change across stimulation for both relaxation and force production, we used R package 'lme4' to construct linear mixed effects models to test for the fixed effect of treatment (stimulation profiles of drum speed and length), with the random intercept of individual ID to address the statistical non-independence of replicates. These tests were then followed by Tukey post-hoc comparisons between 0, 1.5, and 3 SDs above mean drum speed (held at the mean length) or the same treatments in regard to drum length (held at mean speed). As we had independent hypotheses for the speed and length components of the

Journal of Experimental Biology • Supplementary information

drumming display, we performed separate corresponding statistical models for each component. Species comparisons were treated similarly with post-hoc comparisons determining differences in average percent relaxation and change in force production/relaxation between performing each species drums.

To determine whether a tradeoff or synergistic constraint applied to the components of drum speed and length, we generated models comparing average percent relaxation as well as force and relaxation slopes in response to 0, 1.5, and 3 SDs above mean length at each speed treatment (0, 1.5, and 3 SDs above mean speed). Random effects for these models were the same as for the independent speed and length hypotheses. Evidence of a tradeoff would be represented by a decrease in average percent relaxation in response to extending speed or length beyond the mean or a negative slope suggesting diminishing force production or relaxation across a stimulation train when length and speed were extended. If the pattern remained the same as either the speed manipulation at mean drum length or the length manipulation at mean drum speed, then this would provide evidence for no observable tradeoff.

Journal of Experimental Biology • Supplementary information

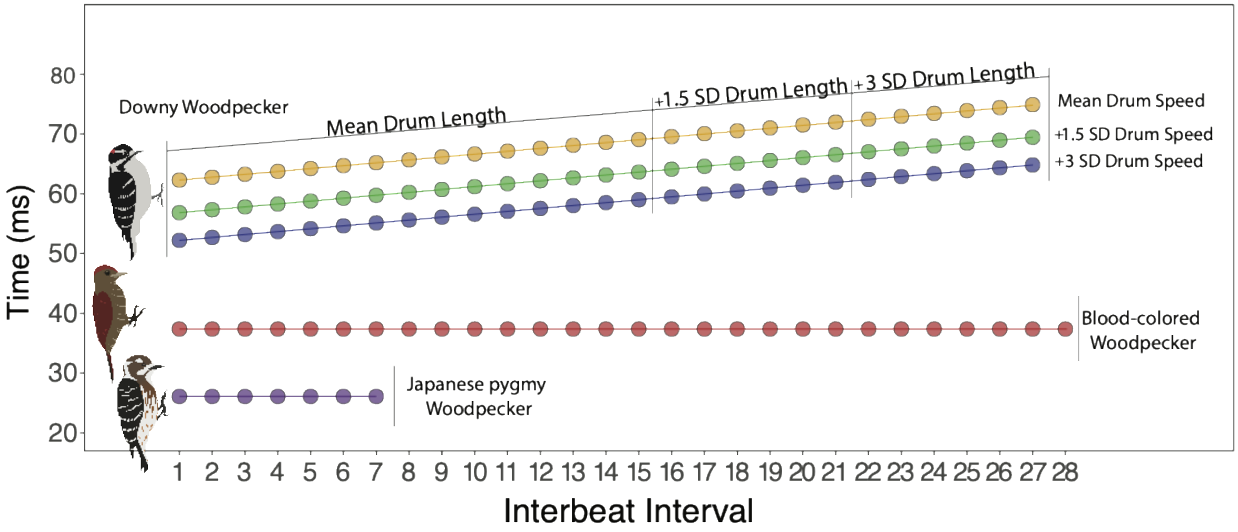

Fig. S1. *Conceptual Framework:* Temporal patterns of each interbeat interval used to generate the stimulation pulses comparing length, speed, and species differences. Roman numerals denote the 5 stimulation pulse sets, with vertical lines representing mean and extended species drum lengths. Woodpecker illustrations by C.F. Stowers.

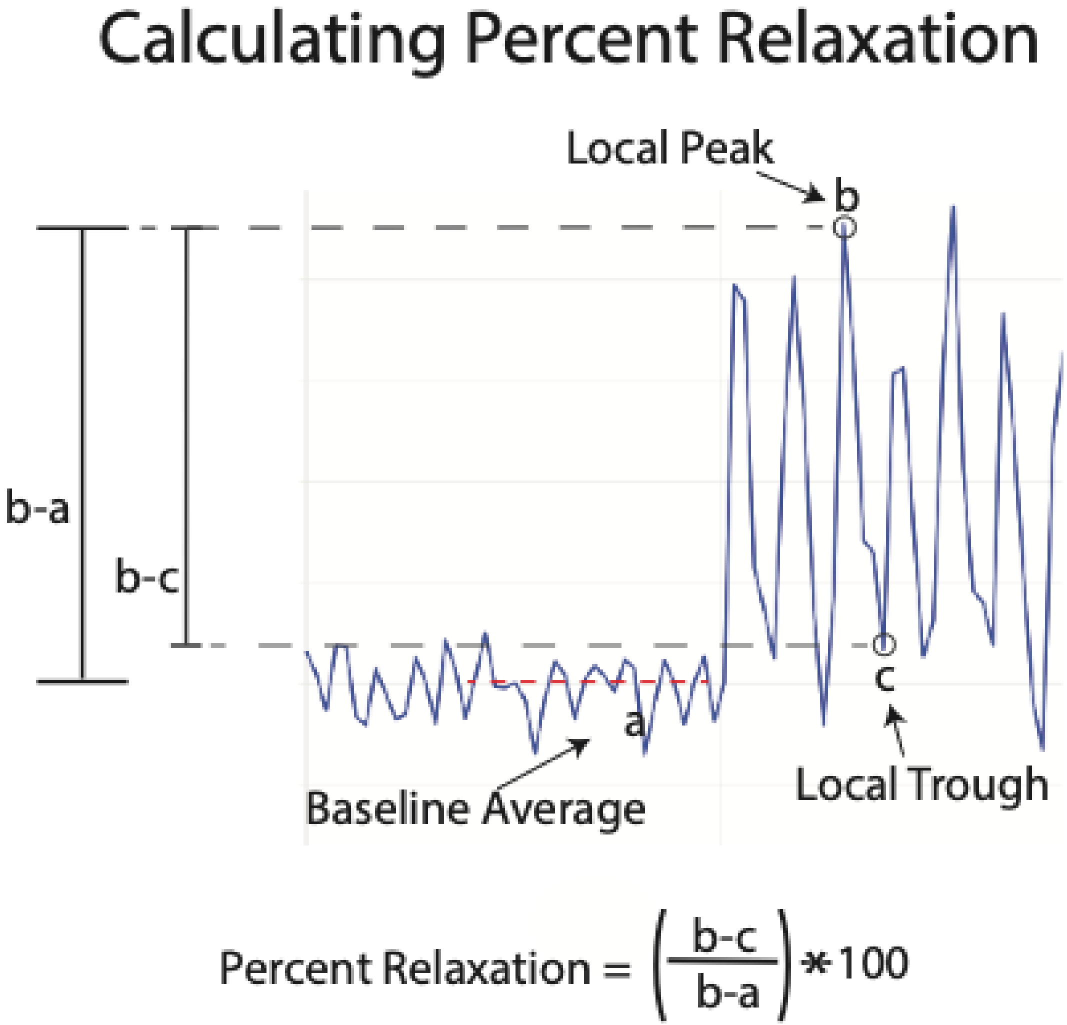

Fig. S2. Force trace schematic showing how percent relaxation was calculated for each twitch cycle. The diagram shows the baseline signal on the force transducer followed by six full twitch cycles. The baseline average is represented by (a) and the example for local peak (b) and trough (c) represent the response to the first pulse of the stimulation train. Thus, (b-c) represents some fraction of (a-c) multiplied by 100 to determine percent relaxation. Axes units are arbitrary in this illustrative example.

Table S1. Tukey post hoc comparisons of speed treatments within each length treatment for percent relaxation. Pairwise contrasts compare estimated marginal means (EMMs) of average percent relaxation between speed treatments (mean speed, +1.5 SD, +3 SD) at each length treatment (mean, +1.5 SD, +3 SD). Shown are denominator degrees of freedom (df), t-values, and adjusted p-values. Across all length treatments, average percent relaxation was significantly lower at +1.5 SD and +3 SD speeds compared to mean speed, while the difference between +1.5 SD and +3 SD speeds was consistently non-significant.

Speed Treatment Posthoc Comparison	Length Treatment	df	t-value	p-value
**Mean Speed — +1.5 SD Speed**	**Mean Length**	**38.0563**	**3.3202**	**0.0055**
**Mean Speed — +3 SD Speed**	**Mean Length**	**38.0624**	**4.0047**	**0.0008**
**+1.5 SD Speed — +3 SD Speed**	Mean Length	38.2163	0.6885	0.7716
**Mean Speed — +1.5 SD Speed**	**+1.5 SD Length**	**38.0462**	**3.2997**	**0.0058**
**Mean Speed — +3 SD Speed**	**+1.5 SD Length**	**38.0513**	**4.4006**	**0.0002**
**+1.5 SD Speed — +3 SD Speed**	+1.5 SD Length	38.1781	1.1349	0.4989
**Mean Speed — +1.5 SD Speed**	**+3 SD Length**	**38.0471**	**3.1643**	**0.0084**
**Mean Speed — +3 SD Speed**	**+3 SD Length**	**38.0523**	**3.5603**	**0.0029**
**+1.5 SD Speed — +3 SD Speed**	+3 SD Length	38.1815	0.3817	0.923

Journal of Experimental Biology • Supplementary information

Table S2. Tukey post hoc comparisons of normalized force slopes between speed treatments within each length treatment. Shown are differences in estimated β coefficients (slopes) from linear mixed models, with denominator degrees of freedom (df), t-values, and adjusted p-values. No significant contrasts were detected, indicating that within-train force slopes did not differ systematically among speed treatments at any length.

Speed Treatment Posthoc Comparison	Length Treatment	df	t-value	p-value
Mean Speed — +1.5 SD Speed	0SD	38.0901	0.0453	0.9989
Mean Speed — +3 SD Speed	0SD	38.0994	1.0148	0.5722
+1.5 SD Speed — +3 SD Speed	0SD	38.3428	1.0377	0.558
Mean Speed — +1.5 SD Speed	1.5SD	38.0675	0.683	0.7748
Mean Speed — +3 SD Speed	1.5SD	38.0747	−0.2607	0.9633
+1.5 SD Speed — +3 SD Speed	1.5SD	38.2586	−1.0199	0.569
Mean Speed — +1.5 SD Speed	3SD	38.0555	0.1648	0.9851
Mean Speed — +3 SD Speed	3SD	38.0615	0.8241	0.6905
+1.5 SD Speed — +3 SD Speed	3SD	38.2131	0.704	0.7626

Journal of Experimental Biology • Supplementary information

Table S3. Estimated slopes (β coefficients) for change in normalized force across stimulation trains under different speed and length treatments. Values are model-based means (± SE) with denominator degrees of freedom (df) and 95% confidence intervals (CI) from linear mixed models. Slopes reflect the within-train trajectory of force production across successive twitches. Only two of the nine speed-length combinations showed consistently negative slopes, and these did not align in a systematic pattern with increasing speed or length, suggesting that force output was generally maintained across trains.

Speed Treatment	Length Treatment	Mean β (Slope)	SE	df	Lower 95% CI	Upper 95% CI
**Mean Speed**	Mean Length	-0.0062	0.0031	8.1482	-0.0134	0.001
**Mean Speed**	+1.5 SD Length	-0.0055	0.0024	7.0336	-0.0113	0.0002
**Mean Speed**	+3 SD Length	-0.003	0.0019	6.4544	-0.0075	0.0016
**+1.5 SD Speed**	Mean Length	-0.0064	0.003	6.6614	-0.0135	0.0008
**+1.5 SD Speed**	**+1.5 SD Length**	**-0.0068**	**0.0023**	**5.9668**	**-0.0126**	**-0.0011**
**+1.5 SD Speed**	+3 SD Length	-0.0032	0.0018	5.6017	-0.0078	0.0014
**+3 SD Speed**	**Mean Length**	**-0.0089**	**0.0029**	**6.3336**	**-0.016**	**-0.0018**
**+3 SD Speed**	+1.5 SD Length	-0.0051	0.0023	5.7221	-0.0108	0.0007
**+3 SD Speed**	+3 SD Length	-0.0041	0.0018	5.4017	-0.0086	0.0005

Journal of Experimental Biology • Supplementary information

Table S4. Estimated slopes (β coefficients) for change in percent relaxation across stimulation trains under different speed and length treatments. Values are model-based means (± SE) with denominator degrees of freedom (df) and 95% confidence intervals (CI) from linear mixed models. Slopes describe the within-train trajectory of relaxation across successive twitches. Confidence intervals generally overlapped zero, indicating no consistently negative trend across treatments that would be indicative of constraint; some conditions even showed small positive slopes. This was likely due to the rhythm of the downy woodpecker, and thus the reflective stimulus program to the muscle, slowing slightly across twitches within a train.

Speed Treatment	Length Treatment	Mean β (Slope)	SE	df	Lower 95% CI	Upper 95% CI
**Mean Speed**	Mean Length	-0.2447	0.1721	12.2125	-0.619	0.1296
**Mean Speed**	+1.5 SD Length	0.0604	0.0888	30.0719	-0.121	0.2418
**Mean Speed**	+3 SD Length	0.019	0.068	30.0719	-0.1199	0.1578
**+1.5 SD Speed**	Mean Length	0.2837	0.1593	9.1471	-0.0757	0.6431
**+1.5 SD Speed**	**+1.5 SD Length**	**0.3566**	**0.078**	**20.7793**	**0.1944**	**0.5188**
**+1.5 SD Speed**	**+3 SD Length**	**0.1895**	**0.0597**	**20.7793**	**0.0654**	**0.3137**
**+3 SD Speed**	Mean Length	-0.159	0.1557	8.5613	-0.514	0.1959
**+3 SD Speed**	+1.5 SD Length	0.1265	0.0745	21.0133	-0.0284	0.2814
**+3 SD Speed**	**+3 SD Length**	**0.2294**	**0.057**	**21.0133**	**0.1109**	**0.348**

Journal of Experimental Biology • Supplementary information

Table S5. Tukey post hoc comparisons of species treatments for percent relaxation. Pairwise contrasts compare estimated marginal means (EMMs) of average percent relaxation between species treatments (Downy, Japanese pygmy, and Blood-colored woodpecker drums). Shown are denominator degrees of freedom (df), t-values, and adjusted p-values.

Species Treatment Posthoc Comparison	df	t-value	p-value
**Downy woodpecker - Japanese pygmy woodpecker**	**37.1197**	**13.741**	**<0.001**
**Downy woodpecker - Blood-colored woodpecker**	**37.0483**	**6.0806**	**<0.001**
**Japanese pygmy woodpecker - Blood-colored woodpecker**	**37.0288**	**-8.0594**	**<0.001**

Table S6. Estimated slopes (β coefficients) for change in percent relaxation across stimulation trains under different species treatments. Values are model-based means (± SE) with denominator degrees of freedom (df) and 95% confidence intervals (CI) from linear mixed models. Slopes describe the within-train trajectory of relaxation across successive twitches. Confidence intervals consistently overlapped zero, indicating no consistently negative trend across species treatments that would be indicative of muscular constraint.

Species Treatment	Mean Slope (β)	SE	df	Lower 95% CI	Upper 95% CI
**Downy woodpecker**	-0.2509	0.3096	16.5717	-0.9054	0.4037
**Japanese pygmy woodpecker**	0.2292	0.273	11.0601	-0.3712	0.8296
**Blood-colored woodpecker**	0.0933	0.2842	12.8589	-0.5214	0.708

Table S7. Tukey post hoc comparisons of normalized force slopes between species treatments. Shown are differences in estimated β coefficients (slopes) from linear mixed models, with denominator degrees of freedom (df), t-values, and adjusted p-values.

Species Treatment Posthoc Comparison	df	t-value	p-value
Downy woodpecker - Japanese pygmy woodpecker	**37.1697**	**-8.6258**	**<0.001**
Downy woodpecker - Blood-colored woodpecker	37.0685	-1.2661	0.4229
Japanese pygmy woodpecker - Blood-colored woodpecker	**37.0409**	**7.852**	**<0.001**

Table S8. Estimated slopes (β coefficients) for change in normalized force across stimulation trains simulating different species drums. Values are model-based means (± SE) with denominator degrees of freedom (df) and 95% confidence intervals (CI) from linear mixed models. Slopes reflect the within-train trajectory of force production across successive twitches. Only the Japanese pygmy woodpecker treatment showed a consistent slope, though it was notably positive, increasing across the stimulation train. Thus, these patterns suggested that force output was generally maintained or even increased across trains.

Species Treatment	Mean Slope (β)	SE	df	Lower 95% CI	Upper 95% CI
Downy woodpecker	-0.0064	0.0051	7.6069	-0.0182	0.0054
Japanese pygmy woodpecker	**0.0292**	**0.0048**	**6.0343**	**0.0175**	**0.0409**
Blood-colored woodpecker	-0.0011	0.0049	6.4973	-0.0127	0.0106

Journal of Experimental Biology • Supplementary information

Supplemental References

Antonson, N. D., Ogunbiyi, S., Champigneulle, M., Roberts, T. J., Goller, F. and Fuxjager, M. J. (2025). Neuromuscular coordination of movement and breathing forges a hammer-like mechanism for woodpecker drilling. *Journal of Experimental Biology*. *In Press*.

Elemans, C. P. H., Mead, A. F., Rome, L. C. and Goller, F. (2008). Superfast Vocal Muscles Control Song Production in Songbirds. *PLOS ONE* **3**, e2581.

Mencio, C., Kuberan, B. and Goller, F. (2017). Contributions of rapid neuromuscular transmission to the fine control of acoustic parameters of birdsong. *Journal of Neurophysiology* **117**, 637–645.

Miles, M. C., Schuppe, E. R., Ligon, R. M. and Fuxjager, M. J. (2018). Macroevolutionary patterning of woodpecker drums reveals how sexual selection elaborates signals under constraint. *Proceedings of the Royal Society B: Biological Sciences* **285**, 20172628.

Miles, M. C., Schuppe, E. R. and Fuxjager, M. J. (2020). Selection for Rhythm as a Trigger for Recursive Evolution in the Elaborate Display System of Woodpeckers. *The American Naturalist* **195**, 772–787.

Schuppe, E. R. and Fuxjager, M. J. (2018). High-speed displays encoding motor skill trigger elevated territorial aggression in downy woodpeckers. *Functional Ecology* **32**, 450–460.

Journal of Experimental Biology • Supplementary information
